# Innovative approaches for induction of gastrointestinal anastomotic healing: an update on experimental and clinical aspects

**DOI:** 10.1007/s00423-020-01957-1

**Published:** 2020-08-15

**Authors:** Stefan Reischl, Dirk Wilhelm, Helmut Friess, Philipp-Alexander Neumann

**Affiliations:** Department of Surgery, School of Medicine, Klinikum rechts der Isar, Technical University of Munich, Munich, Germany

**Keywords:** Anastomotic healing, Anastomotic leak, Intestinal healing, Approach, Treatment, Prophylaxis

## Abstract

**Purpose:**

In most cases, traditional techniques to perform an anastomosis following gastrointestinal resections lead to successful healing. However, despite focused research in the field, in certain high-risk situations leakage rates remain almost unchanged. Here, additional techniques may help the surgeon to protect the anastomosis and prevent leakage. We give an overview of some of the latest developments on experimental and clinical techniques for induction of anastomotic healing.

**Methods:**

We performed a review of the current literature on approaches to improve anastomotic healing.

**Results:**

Many promising approaches with a high clinical potential are in the developmental pipeline. Highly experimental approaches like inhibition of matrix metalloproteinases, stem cell therapy, hyperbaric oxygen therapy, induction of the hypoxic adaptive response, and the administration of growth factors are still in the preclinical phase. Other more clinical developments aim to strengthen the anastomotic suture line mechanically while shielding it from the influence of the microbiome. Among them are gluing, seaming the staple line, attachment of laminar biomaterials, and temporary intraluminal tubes. In addition, individualized bowel preparation, selectively reducing certain detrimental microbial populations could become the next stage of bowel preparation. Compression anastomoses are evolving as an equivalent technique additional to established hand-sewn and stapled anastomoses. Fluorescence angiography and flexible endoscopy could complement intraoperative quality control additionally to the air leak tests. Virtual ileostomy is a concept to prepare the bowel for the easy formation of a stoma in case of leakage.

**Conclusion:**

A variety of promising diagnostic and prophylactic measures that may support the surgeon in identifying high-risk anastomoses and support them according to their potential deficits is currently in development.

## Introduction

Despite technical advancements and focused research, anastomotic healing still fails in up to 20% of cases [1]. Compromised healing results in anastomotic leakage, which is defined as communication between the intraluminal and extraluminal space and thereby may lead to intraabdominal sepsis and death.

The serious consequences of anastomotic leakage have been drawing the surgical scientific scope to this field for decades [2]. Animal studies are the backbone of research, as the complexity of the intestinal healing process cannot be simulated in vitro. While pigs seem to be ideal for studies on surgical techniques, mouse models are probably the model organism of the future due to short breeding times, availability of knockout models, and an intraabdominal immune response comparable with humans [3–7].

Despite methodological deficits, experimental research has drawn a conclusive image of intestinal healing physiology, which will be demonstrated briefly in the following. Intestinal anastomotic healing is classically divided into three phases. In the first, the inflammatory phase, hemostasis and preliminary spanning of the gap between the wound edges takes place. The hemostatic clot forms a matrix, which is further immigrated by immune cells to form the inflammatory infiltrate. In this phase a timed shift from pro- to anti-inflammatory signaling is important to restrict the necessary inflammatory response to a physiological limit. This concept of the resolution of inflammation is marked by a phenotypical switch of immune cells. In the following proliferative phase (myo)fibroblasts migrate to the healing tissue, proliferate, and induce collagen formation. From this point, anastomotic stability is mediated by a stable layer of collagen, and the sutures lose importance. Still, full mechanical stability is restored later in the reparative phase, by turnover and remodeling of the collagen type and fibers to form a stable, functional scar. Several molecular classes as growth factors, interleukins, and chemokines mediate communication between immune cells and matrix forming cells. Collagen degrading enzymes, so-called matrix metalloproteinases (MMP), are key players highly active in the early healing phases [8]. Their balanced regulation is important to allow reorganization of collagen without endangering the integrity of the newly formed collagen layer. In the colorectum the microbiome is an additional component of high relevance. Certain microbial stems have the potential to directly increase MMP activity and thereby impair closure of the defect [9], while other populations seem to have protective functions on anastomotic healing by preserving microbial homeostasis [10].

Although a lot of research focuses on unraveling the healing physiology and the relevant molecular players are identified, far less is known about why anastomotic healing fails in certain cases [11]. Clinical experience suggests that the early healing phase is most endangered. Still, also in the later phases, gap formations in the healing tissue can occur, leading to fistulas and intraabdominal abscesses, which usually cannot be cured without further interventions. Even if a technically insufficient suture with primary gap formation can be avoided, there are many possible conditions to compromise the necessary balance in the healing process: colitis, peritonitis, immunosuppression, radiation, chemotherapy, diabetes, and lack of blood supply. Basing on that knowledge a broad variety of treatment approaches has been developed at different stages of the translational process. In the following, we want to demonstrate innovative experimental approaches with special emphasis to their stage of translation but also give a brief update on the evidence of standard techniques to improve anastomotic healing.

## Experimental approaches for induction of anastomotic healing

**Fig. 1:**
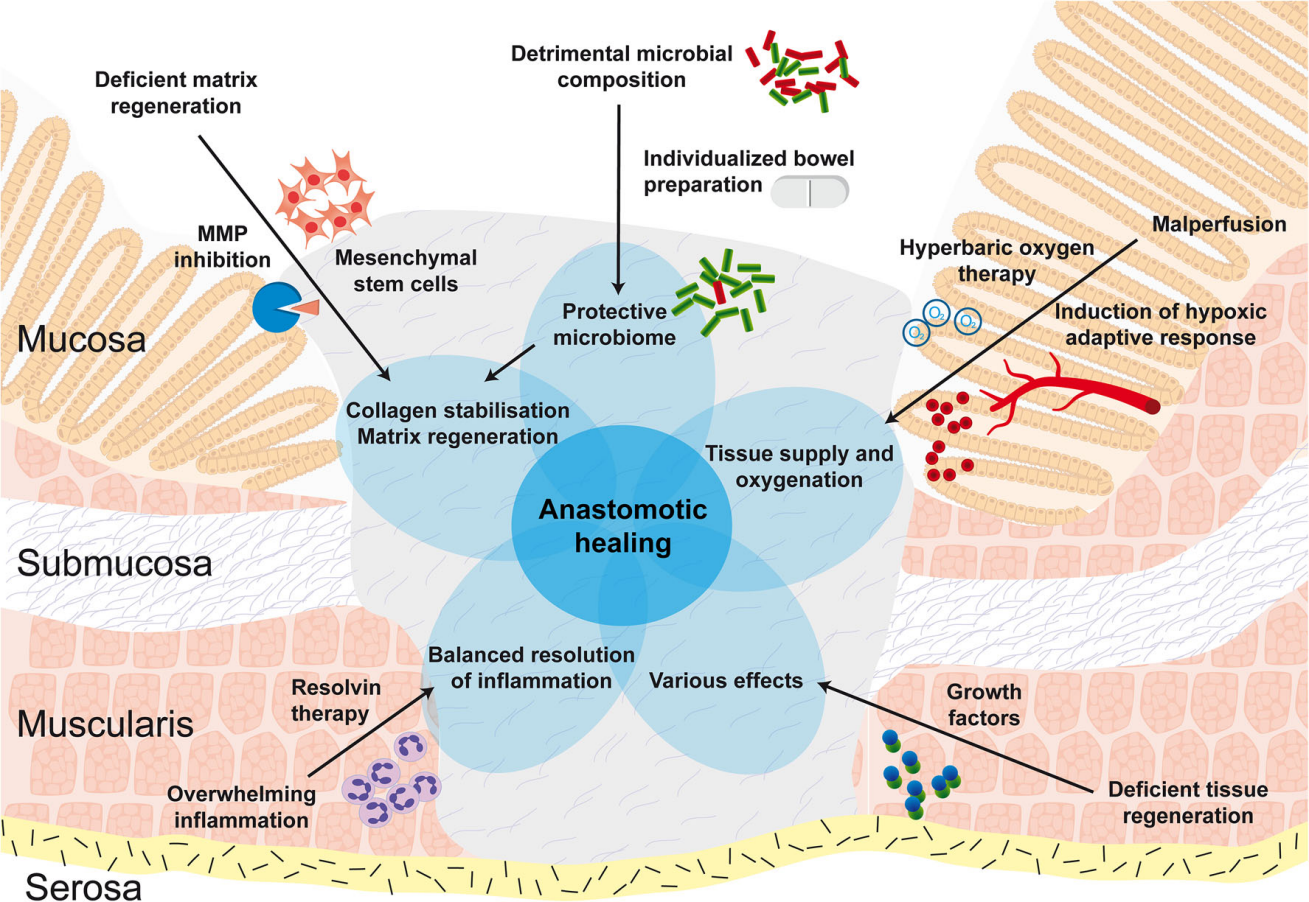
Innovative experimental approaches to improve gastrointestinal anastomotic healing. Various approaches to improve anastomotic healing have been developed to different stages in the translational process. This figure demonstrates the mainly experimental approaches. Among them are stem cell therapy, individualized bowel preparation, hyperbaric oxygen therapy and induction of the hypoxic adaptive response, matrix metalloprotease inhibition, growth factor administration, and anti-inflammatory therapies

The most innovative experimental approaches try to influence certain steps of the healing process and thereby directly aim to improve the healing physiology (Fig. 1). Most of these are pharmacological approaches and still in early preclinical phases. Although it is not possible to forecast the future significance of an approach, we will try to give an impression of the individual potential.

### Influence of the intestinal microbiome on anastomotic healing: Individualized bowel preparation

We are just at the beginning of understanding the complex implications of the microbiome on anastomotic healing. Current studies suggest that distinct bacterial populations (e.g., *Enterococcus faecalis*, *Bacillus subtilis*) have the ability to degrade collagen and thereby endanger anastomotic healing [9, 10]. On the other hand, obligate anaerobes such as *Clostridium coccoides*, *Bacteroides fragilis*, *Bifidobacterium* *spp.*, and *Prevotella* *spp.* seem to be important in maintaining gastrointestinal homeostasis [12] and could be beneficial in anastomotic healing. Additionally, surgery itself influences the composition of the microbiome [13].

Oral antibiotic bowel preparation is only performed in 15% of elective colorectal operations in Germany [14], mostly with a combination of broad-spectrum antibiotics such as neomycin and metronidazole. Although oral antibiotics but not mechanical bowel preparation additional to preoperative intravenous antibiotic treatment had a positive effect on surgical site infections and mortality in current meta-analyses [15–19], there was no effect of oral antibiotic bowel preparation on anastomotic leakage rates. In summary, preoperative intravenous antibiosis and oral antibiotic bowel preparation are recommended, as they reduce postoperative complications, although being an imprecise approach with no effect on anastomotic leakage. This gap could be filled by preoperative analysis of the microbial composition and individualized bowel preparation, either by specific antibiotics or nutrients, leading to an optimization of the microbial balance finally improving anastomotic healing [20]. Still, that approach is at a very early stage of the developmental process and will need further evaluation of its effectiveness. Further research on the influence of the microbiome may shed more light on its potential use in the clinic.

### Matrix metalloproteinase inhibition

The significance of MMPs in anastomotic healing has been known since the 1990s [21, 22]. Those enzymes have the capacity to degrade collagen and thereby endanger the stability of the early anastomotic closure. It has been shown that immediately after surgery the MMP activity is markedly upregulated [8]. In some early animal studies in rats, unselective MMP inhibition could improve bursting pressures [23]. Specific MMP inhibition was shown to reduce leakage rate and improve bursting pressures, recently [24]. Although there are no intervention studies in human patients yet, inhibition of MMPs may be a promising approach to prevent leak formation. However, it has to be taken into account that MMP activity is absolutely necessary for the anastomosis to heal. Successful inhibition of MMPs for prevention of leakage formation needs to be well timed and is therefore a highly complex approach.

### Growth factors

The use of growth factors to induce gastrointestinal healing processes is quite an inhomogeneous field of experimental research, that has shown some beneficial effects, but is hard to be generalized [11, 23]. Among the potential candidates are insulin-like growth factor I (IGF-1), growth hormone (GH), fibroblast growth factor (FGF), epidermal growth factor (EGF), heparin binding EGF-like growth factor, transforming growth factor β (TGF- β), vascular endothelial growth factor (VEGF), and platelet-derived growth factor (PDGF). Most experimental evidence is available on IGF-1 and GH and shows positive effects on anastomotic healing, but the heterogeneity of experimental studies is enormous. Actually, the next step in translation is clinical studies. Still, the possible danger of using mitogenic substances in cancer patients might disqualify these agents from further exploration in clinical trials. That risk has to be examined in further animal studies or alternatively the substances could be delivered locally to circumvent the risk of harm [23].

### Hyperbaric oxygen therapy

Hyperbaric oxygen therapy (HBOT) is the administration of 100% oxygen at 2–3 times atmospheric pressure, which requires allocation of the patient to a hyperbaric chamber postoperatively for several times. Although this therapy could improve anastomotic healing in a number of experimental studies in rats, the studies are to inhomogeneous to derive valid conclusions [25, 26]. Furthermore, no human studies were performed yet. According to the high apparative effort of HBOT, the practicability and future role remains elusive.

### Induction of the hypoxic adaptive response

A feasible approach considering the oxygen metabolism could be the induction of the hypoxic response. Erythropoietin (EPO) and vascular endothelial growth factor (VEGF) are induced as mediators of the adaptive response to hypoxia. The combination is potent to induce immediate tissue protection and inducing angiogenesis to improve the oxygen supply in ischemic tissues in the medium-term. Thus, the approach of their pharmacological administration could be particularly promising for anastomoses of questionable perfusion. Still, there is very few preclinical, yet positive evidence on administration of EPO and VEGF to improve anastomotic healing [11, 23, 27]. Lack of known side-effects of short time treatment would allow perioperative treatment. Still, future significance is unclear as other experimental approaches showed stronger effects.

### Cellular therapy

A novel option, first described 10 years ago is the administration of mesenchymal stem cells (MSC) or bone marrow–derived mononuclear cells (BM-MNC), alternatively. Mesenchymal stem cells are isolated from different sources (mostly adipose tissue or bone marrow), which have to be cultured prior to injection due to low numbers. In contrast, BM-MNCs can be retrieved from the bone marrow at high concentrations and can be administered without further preparation. There is only a very limited number of preclinical studies showing first promising results [28–35]. Still, stem cells have not been examined in clinical studies with the intention to improve anastomotic healing so far. Experimental studies in rat and porcine models show some promising results of topic or systemic administration, especially in high risk anastomoses, such as ischemia or colitis. One major problem of cellular therapy is difficulty of standardization in the isolation process, which leads to high variations of therapeutic cellular material [36]. Thus, experimental results have to be interpreted with even more caution. In conclusion, the future role of stem cell therapy to induce healing processes in the intestine has to be determined and is still far away from clinical use. Additionally, some studies raise concerns that stem cells could also migrate to malignant tumors and promote tumor growth in various tumor types including colorectal cancer [37, 38].

### Anti-inflammatory treatment

Non-steroidal anti-inflammatory drugs (NSAIDs) are currently routinely used as part of the postoperative analgesic treatment schematic. Current meta-analyses identified a detrimental effect of postoperative NSAID treatment on anastomotic healing, which seems to be mainly mediated by non-selective NSAIDs [39, 40]. The negative effect on anastomotic healing was pronounced in colorectal anastomoses, non-selective NSAIDs, and protocoled use compared with sporadic use. Those results suggest that the intrinsic inflammatory response is a necessary prerequisite for anastomotic healing and its complete suppression impairs anastomotic healing. Therefore, strategies promoting the resolution of inflammation, while allowing the initial inflammatory response, could be promising therapeutic regimens. Especially in cases with overwhelming mucosal inflammation (e.g., intestinal surgery during colitis) this approach might be useful. There is an emerging role of specific proresolving mediators in several chronic inflammatory disease types (e.g., arthritis and cardiovascular disease) [41, 42]. Still, the use of those drug classes has not been used to improve anastomotic healing yet, but should be taken into account in the future.

## Protective measures to prevent anastomotic leakage

**Fig. 2:**
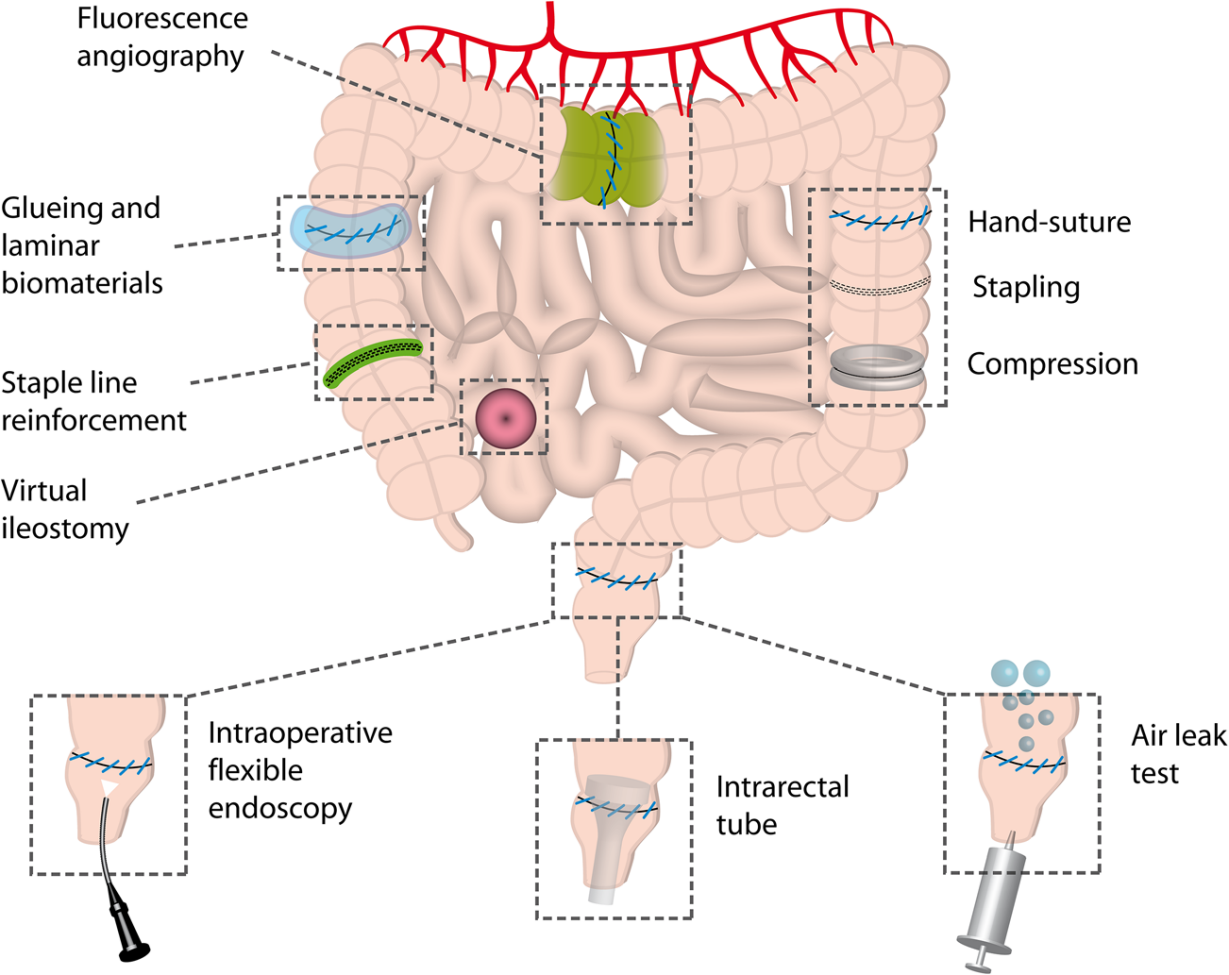
Technical approaches to improve gastrointestinal anastomotic healing and predict anastomotic leakage. This figure shows mainly clinical and technical approaches and diagnostic methods to assure the quality of the anastomosis intraoperatively. Hand-suture, stapling, and compression are equal technical approaches with some individual features. Diagnostic methods comprise fluorescence angiography, flexible endoscopy, and the air leak test. Some methods aim at additional shielding of the anastomosis like gluing, laminar biomaterial use, or staple line reinforcement and intraluminal tubes. Virtual ileostomy aims to prepare easy stoma formation in case of anastomotic leakage

Apart from improving healing physiology and bowel preparation described above, many strategies aim to protect the anastomosis from mechanical or infectious stress (Fig. 2). A lot of effort has been spent on examination of possibilities to additionally protect intestinal anastomoses after suturing or stapling. Most of the concepts are based on shielding the anastomosis from the detrimental influence of the microbiome, particularly in the colorectum (e.g., stoma formation, transanal tubes, staple line reinforcement). Some strategies additionally aim at mechanical strengthening of the suture (e.g., gluing).

### Surgical gluing and covering with laminar biomaterials

Surgical gluing was initially used to glue superficial wounds and was considered for intestinal use later. Most studies are focusing on cyanoacrylate and fibrin glue. Cyanoacrylate was developed in 1948, served as industrial glue initially, but was soon admitted as surgical glue due to its ability to stop bleeding [43, 44]. Fibrin glue is basically a two-component glue of fibrinogen and thrombin, sometimes with additional supplements as fibronectin [45]. The rationale is convincing at a first glance, as mechanical strengthening and microbial shielding could be addressed by these approaches.

Both gluing strategies were examined in many experimental and few human studies of low quality. The existing data on glues are inconclusive, although promising. While fibrin glue seems to have positive effects on anastomotic healing in gastric, ileal, and colonic anastomoses, it seems to be superior to cyanoacrylate particularly in the colon [46]. The superiority of fibrin glue to cyanoacrylate could be explained by the physiological properties of fibrin glue, being resorbable. Still, in the animal models fibrin glue did not improve the healing process itself, so the positive effects are rather by mechanical strengthening or sealing [47]. In summary, high-quality clinical studies are necessary, preferably focusing on physiological glues, for example fibrin- or collagen-like formulations.

Another concept, taking the idea of gluing even further, is the additional attachment of laminar biomaterials as Tachosil (Takeda, Tokyo, Japan) on the completed anastomosis [48–50]. Although study quality is quite poor and consistent positive effects could not be found, the concept of compartmentalization between intraluminal and extraluminal space could be promising and may not be abandoned too soon. Particularly combination with glues could improve adhesion properties. Still, the problem of microbial colonization of biocompatible materials is a problem. It may be approached by antibiotic or antiseptic loading of the materials, which is already a common strategy in orthopedic, but not in visceral surgery [51, 52].

### Staple line reinforcement

Stapling is a discontinuous way of connecting the tissue with small gaps between the staples, although multiple offset staple lines are already minimizing that problem in modern stapling devices. Still, staple line reinforcement strategies have been developed, that are attached to the stapler and stapled between the tissue layers during stapling. For example, the Seamguard (Gore, Newark, US) staple line reinforcement is a bioabsorbable laminar matrix consisting of polyglycolic acid and trimethylene carbonate, which is stapled between the intestinal layers for mechanical buttressing. There are some clinical studies of poor quality, mainly focusing on the use in gastric/bariatric and less in colorectal anastomoses [53–59]. Although it was safe and feasible in those studies, no consistent positive effect on anastomotic healing could be determined so far [56, 58, 60].

### Intrarectal tube devices

An evolution of stoma formation could be presented by mechanical intraluminal protection of the (colo)rectal anastomosis by tubes attached above or at level of the anastomosis. Current meta-analyses show a beneficial effect of small lumen transanal decompression tubes on anastomotic leakage [61–65] but include only one randomized controlled trial (RCT) [66]. Another entity are flexible tube-like intraluminal devices with a higher diameter. There is a broad variety of approaches: Coloshield, C-seal, Valtrac-secured intracolonic bypass, Korean fecal diverting device, Cologuard, and Colovac. Although the principle is the same, they differ in materials and way of fixation. Most positive evidence is from animal studies or observational studies. Only two RCTs have been performed: one on the C-seal [67], showing disastrous results with a leakage rate double of the control group, and one on the Korean fecal diverting device [68], which showed equality to stoma formation, but had methodological deficits. In summary, intracolonic bypassing could be a promising approach for rectal anastomoses but is still not widely used in the clinical practice and waiting for a breakthrough. One can only speculate on the reasons: Medical devices can usually only be successfully introduced by companies who can organize an optimal marketing campaign, which is not the case for those devices. Furthermore, the application is time-consuming and demanding [69]. Still, although the existing devices were rather disappointing, the concept should not be abandoned yet.

### Stoma and virtual ileostomy

Defunctioning stoma has a robust value in low anterior rectal resections to reduce the consequences of postoperative anastomotic leakage [70–74]. A novelty, trying to evolve the idea of ileostomy while reducing the disadvantages, is virtual or ghost ileostomy. A bowel loop proximal to the ileocecal valve is marked and approximated to the bowel wall by a vessel loop [75]. It can be easily converted into a real ileostomy in case of anastomotic dehiscence, but be removed in case of regular healing. In a recent review of a total number of 11 studies virtual ileostomy qualifies as a safe procedure, with a low complication rate of SSI, hernias, and twisting [75]. Conversion was only necessary in 10% of cases. Still, it is not clear if patients with primary stoma have a better outcome, than patients with an ileostomy converted from a ghost ileostomy after manifestation of anastomotic leakage.

## Comparison of conventional techniques

### Suture techniques

The indispensable prerequisite for anastomotic healing seems to be the flawless connection of the bowel ends after resection, as without it, healing cannot occur. Over the years various surgical methods and materials for mechanical approximation of the wound edges after intestinal resection were developed. Suturing was the initial method [76]. Still, at an early stage the compression anastomosis was already described by Denan in 1826 but then abandoned for a long time [77–80]. Surgical stapling devices entered the stage quite late and were used from the 1970s. At the moment, stapled and hand-sewn anastomoses are both widely used, while the compression anastomosis is rather a rarity.

Level 1a evidence indicates that stapling and hand-sewn anastomoses give equal results with regard to clinical anastomotic leakage, although hand-sewn anastomoses tend towards longer operation times [81–91]. For compression anastomoses there is a variety of devices and methods. Today the biofragmentable anastomotic ring, which leaves the colon via the natural way after a few days, is used predominantly. In 2006 the NiTi CAR 27 ring was introduced, which should guarantee consistent compression by nitinol springs. Newer methods are the CARP (compression anastomotic ring-locking procedure) [92] and the so-called Magnamosis by magnetic rings [93]. At a first glance, compression anastomoses could have advantages due to constant pressure distribution, avoiding local nutrient and blood undersupply, lack of gap formation, and foreign body reactions to staples or sutures. A meta-analysis comparing 10 RCTs of compression anastomoses to conventional technique (hand-sewn or stapled anastomoses) showed equality of the compression anastomosis to the conventional techniques in terms of leakage rates [84]. Still, colorectal compression anastomoses had a significantly shorter time to return of bowel function, while the obstruction rate was higher.

In summary, no general recommendation for one of the three techniques can be given, as all types are safe. Therefore, the selection may be dependent from the surgeon’s preferences and abilities and the technical feasibility in the intended anastomotic location. Still one can find certain differences to the hand-sewn anastomosis: shorter operative time and higher costs in stapled and compression anastomoses, potential detrimental effects on obstruction in compression anastomoses, and higher postoperative bleeding rates in stapled anastomoses. Hence, special attention should be spent to those issues during the operation. Stapling and compression devices are particularly appropriate for distal colorectal anastomoses, as the device can be introduced through the anus and does not require an additional intestinal incision. In summary, the hand-sewn anastomosis will even in the future be the baseline technique, which every visceral surgeon has to master, as it can be adapted to all situations. If the tissue seems appropriate, compression or stapled anastomoses can be used as standardized connection techniques.

## Intraoperative quality control

Additional to prophylactic measures to protect the anastomosis, surgeons need diagnostic tools to identify anastomoses at risk. Surgeons are currently not able to predict which anastomoses will leak and which will not, although most are certain that they can [94]. Additional to the experience of the surgeon to judge the quality of the completed anastomosis basing on macroscopical hints (e.g., signs of ischemia, macroscopic leaks, fat tissue in the stapler line) several tests for intraoperative quality control of the anastomosis are used (Fig. 2). Those tests are either examining the tightness of the suture line or the blood supply.

### Air leak test

The baseline test for tightness of the anastomosis is the air leak test. In principle, the rectum is filled with air from the anus after completion of the suture, while the situs is filled up with irrigation solution. If any air passes the anastomosis, the air leak test is positive. Depending on the severity of the insufficiency a revision of the anastomosis should be considered. A meta-analysis found no difference in the risk of anastomotic leak between patients with or without intraoperative air leak test [95]. Still, the rate of anastomotic leak was significantly higher in the group with an initial positive air leak test, although almost all anastomoses were revised intraoperatively in that group [95]. Additionally, the only included RCT found a significant higher risk for anastomotic leak, if no air leak test was performed [96]. Hence, the air leak test can be recommended for distal colorectal anastomoses as it is economic and has a good predictive value.

### Fluorescence angiography

Fluorescence angiography is a procedure to monitor perfusion of the tissue via optical detection of a fluorescent indocyanine dye injected intravenously. The dye itself is cheap (~ 15 euros), while the necessary fluorescence microscope is an expensive apparatus (~ 100,000 euros). A meta-analysis of six case-control studies could show reduced anastomotic leakage rates by usage of intraoperative fluorescence angiography [97]. Patients with revision of the transection line due to malperfusion in indocyanine fluorescence examination still had higher anastomotic leakage rates, than patients without revision. Probably that patient collective had comorbidities, globally impairing anastomotic healing. Still, the procedure can be recommended, as it has low running costs, once it is available.

### Intraoperative flexible endoscopy

Intraoperative flexible endoscopy is a method to evaluate the anastomosis from the intestinal lumen. It may be equal to the air leak test for detection of dehiscence, but superior for the detection of bleeding which is a particular problem of stapled anastomoses. One meta-analysis examining six case–control studies detected an advantage of intraoperative endoscopy regarding anastomotic bleeding and postoperative leakage rates [98]. Still, controlled clinical trials are necessary to clarify the significance of intraoperative flexible endoscopy.

## Conclusion

Although anastomotic healing may be successful in many cases, there is a huge need to identify high-risk anastomoses and optimize the available techniques to avoid leakage in those cases. This should consider classical and new risk factors—ischemia and tension, but also malnutrition, inflammation, and the microbiome. Collaborations between universities and companies to transfer the knowledge, finance the development, and guarantee the distribution is necessary to successfully run through the developmental process. This could lead to the next generation of intestinal anastomosis creation—the “anastomosis 2.0”: A broad variety of measures that support the surgeon in identifying high-risk anastomoses and fortify them according to the potential deficits. Even combinations of experimental with technical approaches could be promising (e.g., antiseptic covered suture materials). Furthermore, measures to amend anastomoses that show signs of leakage could further improve outcomes. This may finally lead to the ultimate goal of reducing the incidence of anastomotic leakage to a minimum.
